# Development of an interpretable machine learning model for predicting sarcopenia in patients undergoing maintenance hemodialysis

**DOI:** 10.3389/fmed.2025.1576081

**Published:** 2025-11-04

**Authors:** Shuqin Liu, Xingyu Zhu, Zhixin Wang, Wenwu Tang, Ying Zhang, Huaming Xian, Mi Li, Xisheng Xie

**Affiliations:** ^1^Department of Nephrology, Nanchong Central Hospital Affiliated to North Sichuan Medical College, Nanchong, China; ^2^Department of Nephrology, Guangyuan Central Hospital, Guangyuan, China

**Keywords:** interpretable machine learning, logistic regression model, maintenance hemodialysis, sarcopenia, SHAP analysis

## Abstract

**Background:**

Sarcopenia has a high incidence among patients undergoing maintenance hemodialysis (MHD), significantly increasing the risk of falls, fractures, and mortality. Traditional diagnostic methods, however, are costly and complex, limiting their widespread clinical application. Therefore, developing an efficient and interpretable sarcopenia prediction model using routine clinical and laboratory data is crucial, with explainability techniques applied to further enhance model transparency.

**Methods:**

This study included 256 MHD patients and developed five machine learning models based on clinical and laboratory data: Logistic Regression, Extreme Gradient Boosting, Random Forest, Support Vector Machine, and Gaussian Naive Bayes. Model performance was assessed using the area under the receiver operating characteristic curve (AUC), calibration curve, and decision curve analysis. Additionally, SHapley Additive exPlanations (SHAP) were employed as an explainability tool to enhance and visualize the interpretability of the optimal model.

**Results:**

The Logistic Regression model demonstrated the best performance on the validation set (AUC = 0.828, 95% CI: 0.626–0.989). Key predictive factors included body mass index (BMI), age, gender, creatinine (Cr), 25-hydroxyvitamin D3, left ventricular ejection fraction (LVEF), and estimated glomerular filtration rate (eGFR). SHAP analysis revealed that high BMI and 25-hydroxyvitamin D3 levels were protective factors, while low Cr, LVEF, and eGFR levels, as well as female gender, significantly increased the risk of sarcopenia.

**Conclusion:**

This study developed a Logistic Regression model using an interpretable machine learning approach, offering an efficient tool for early screening of sarcopenia risk in MHD patients and facilitating personalized intervention strategies. However, the single-center design limits the model’s external applicability, and further multi-center studies are necessary to validate its generalizability.

## 1. Introduction

Sarcopenia (SP) is a syndrome characterized by a decline in skeletal muscle mass and function (1), and its incidence is significantly higher in patients undergoing maintenance hemodialysis (MHD) (2). Due to long-term nutritional deficiencies, chronic inflammation, and metabolic disturbances, MHD patients are at an increased risk of developing sarcopenia. This condition not only significantly reduces the quality of life in MHD patients but also increases the risk of falls, fractures, cardiovascular events, and mortality (3, 4). Therefore, early identification and precise intervention for sarcopenia are critical for improving the long-term prognosis of MHD patients.

Currently, the diagnosis of sarcopenia primarily relies on dual-energy X-ray absorptiometry (DXA) to assess muscle mass and handgrip strength testing to evaluate muscle strength (5). Although these methods are relatively accurate, the high cost of equipment and complex procedures limit their widespread application in routine clinical practice. Additionally, a single diagnostic indicator is insufficient to fully capture the complex pathological mechanisms of sarcopenia. Thus, there is an urgent need for an efficient predictive tool that integrates multidimensional clinical and laboratory features.

In recent years, machine learning (ML) technologies have increasingly been applied in disease risk prediction. Their powerful data processing and pattern recognition capabilities offer promising opportunities for the early screening of sarcopenia (6). However, traditional ML models are often considered “black-box” systems, limiting their clinical applicability (7). In contrast, interpretable machine learning (IML) approaches, supported by explainability techniques such as SHapley Additive exPlanations (SHAP), not only enhance transparency but also provide intuitive insights into the contribution of each feature to the prediction, offering valuable decision support for clinicians (8).

While some studies have explored the risk factors for sarcopenia (9), research on developing XML-based sarcopenia prediction models specifically for MHD patients remains limited. This study aims to develop and validate a sarcopenia risk prediction model incorporating SHAP analysis by integrating routine clinical indicators and laboratory data. By combining multidimensional data, this study not only facilitates efficient screening for sarcopenia but also provides scientific evidence to support the development of personalized intervention strategies, ultimately improving the long-term prognosis of MHD patients.

## 2 Materials and methods

### 2.1 Study design and population

This study is a single-center, retrospective observational study conducted at Nanchong Central Hospital from January 2024 to January 2025. A total of 364 MHD patients were included, based on the following inclusion and exclusion criteria: (1) Inclusion Criteria: Age > 18 years, undergoing regular hemodialysis for more than 3 months, and receiving at least 2 dialysis sessions per week. (2) Exclusion Criteria: A history of pacemaker implantation, malignant tumors, kidney transplantation, or amputation; acute infection; incomplete clinical data; or refusal to participate.

### 2.2 Sarcopenia diagnosis criteria

Sarcopenia was diagnosed based on the updated 2019 consensus from the Asian Working Group for Sarcopenia (AWGS) (10), using the following three criteria: (1) Muscle Mass: For men, skeletal muscle mass index (SMI) < 7.0 kg/m^2^; for women, SMI < 5.7 kg/m^2^. (2) Muscle Strength: For men, handgrip strength < 28 kg; for women, handgrip strength < 18 kg. (3) Functional Performance: 6-meter walking speed < 1.0 m/s.

Sarcopenia was diagnosed if a patient met the “muscle mass” criterion and either the “muscle strength” or “functional performance” criterion. To minimize the impact of dialysis-related hydration changes on the results, all measurements were taken post-dialysis.

### 2.3 Data collection and variables

A total of 34 clinical and laboratory variables were collected to comprehensively capture nutritional status and biochemical profiles, which were categorized as follows: (1) General Information: Gender, age, body mass index (BMI), handgrip strength, 6-meter walking speed, dialysis duration, and medical history (hypertension, diabetes, cardiovascular diseases). (2) Body Composition and Functional Indicators: SMI and left ventricular ejection fraction (LVEF). (3) Dialysis Adequacy and Renal Function Indicators: Urea clearance index (Kt/V) and estimated glomerular filtration rate (eGFR). The eGFR was calculated using the CKD-EPI cystatin C equation (2012) as follows: 133 × min (Scys/0.8, 1)^–0^.^499^ × max (Scys/0.8, 1)^–1^.^328^ × 0.996^Age^ × 0.932 (if female), where Scys is serum cystatin C (mg/L) (11). (4) Laboratory Biochemical Indicators: Creatinine (Cr), cystatin C (Cys-C), uric acid (UA), hemoglobin (Hb), albumin (ALB), prealbumin (PA), parathyroid hormone (PTH), neutrophil-to-lymphocyte ratio (NLR), high-sensitivity C-reactive protein (hs-CRP), alkaline phosphatase (ALP), creatine kinase (CK), 25-hydroxyvitamin D3 (25(OH)D3), glucose (GLU), triglycerides (TG), total cholesterol (TC), high-density lipoprotein cholesterol (HDL-C), low-density lipoprotein cholesterol (LDL-C), calcium (Ca), phosphorus (IP), magnesium (Mg), and carbon dioxide (CO2CP).

### 2.4 Model development and validation

#### 2.4.1 Feature selection

Key features were first selected based on Pearson correlation analysis (using the scipy 1.11.3 package, Python), with a threshold of |r| > 0.7 to identify highly correlated variables. The variance inflation factor (VIF) for each variable was then calculated, and variables with a VIF greater than 5 were excluded to mitigate multicollinearity. Subsequently, Least Absolute Shrinkage and Selection Operator (LASSO) regression was applied (with the glmnet package in R software) to further select features. Finally, multivariate logistic regression was performed (using SPSS 27.0) to select significant features with a *p*-value < 0.05 included in the model.

#### 2.4.2 Dataset splitting

The dataset was randomly divided into a training set (*n* = 179) and a validation set (*n* = 77) at a 7:3 ratio, using randomization (performed with Python, scikit-learn library). This first split allowed for model selection and evaluation during the training phase.

Stage 1 (Model Selection): Multiple machine learning models were trained on the training set and evaluated using the validation set. The best-performing model was selected based on its overall performance across multiple evaluation metrics.

Stage 2 (Final Model Training and Independent Testing): After selecting the optimal model, a second split was performed to create an independent test set (*n* = 39, 15% of the total dataset) that was not involved in the model selection process. The final model was retrained using the remaining data (*n* = 217) and its performance was evaluated on this independent test set.

#### 2.4.3 Model construction

Five machine learning models were constructed and compared using Python (with the xgboost, scikit-learn, and matplotlib libraries): Extreme Gradient Boosting (XGBoost), Logistic Regression, Random Forest, Gaussian Naive Bayes (GNB), and Support Vector Machine (SVM). Model performance was assessed using 10-fold cross-validation, with the area under the receiver operating characteristic curve (AUC) as the primary evaluation metric for model accuracy (12).

#### 2.4.4 Model performance evaluation

The calibration and clinical applicability of the models were evaluated using calibration curves, decision curve analysis (DCA), and precision-recall (PR) curves (13–15). Learning curves were plotted to analyze the model’s fitting on both the training and validation sets (16). To generate the learning curves, the proportion of training data was incrementally increased from 10 to 100%, in 10% steps. For each training subset, 10 repetitions of 10-fold cross-validation were performed, with AUC as the primary evaluation metric. The mean AUC (± SD) across all repetitions was calculated, and the 95% confidence intervals were determined using the t-distribution. The curves were plotted with the mean AUC values, and shaded areas representing the confidence intervals helped to visualize the model’s generalization ability and stability.

#### 2.4.5 Model interpretation

SHAP was employed for the interpretability analysis of the optimal model, offering visual insights into feature importance ranking, the contribution of each feature to individual sarcopenia risk, and personalized risk assessments (17).

### 2.5 Statistical analysis

All statistical analyses were performed using SPSS 27.0, R 3.6.1, and Python 3.4.3. Continuous variables were expressed as median and interquartile range (IQR), and group comparisons were conducted using the Mann-Whitney U test. Categorical variables were expressed as frequencies and percentages, with group comparisons made using the chi-square test. A two-tailed *p*-value of < 0.05 was considered statistically significant.

## 3 Results

### 3.1 Basic characteristics of the study population

**FIGURE 1:**
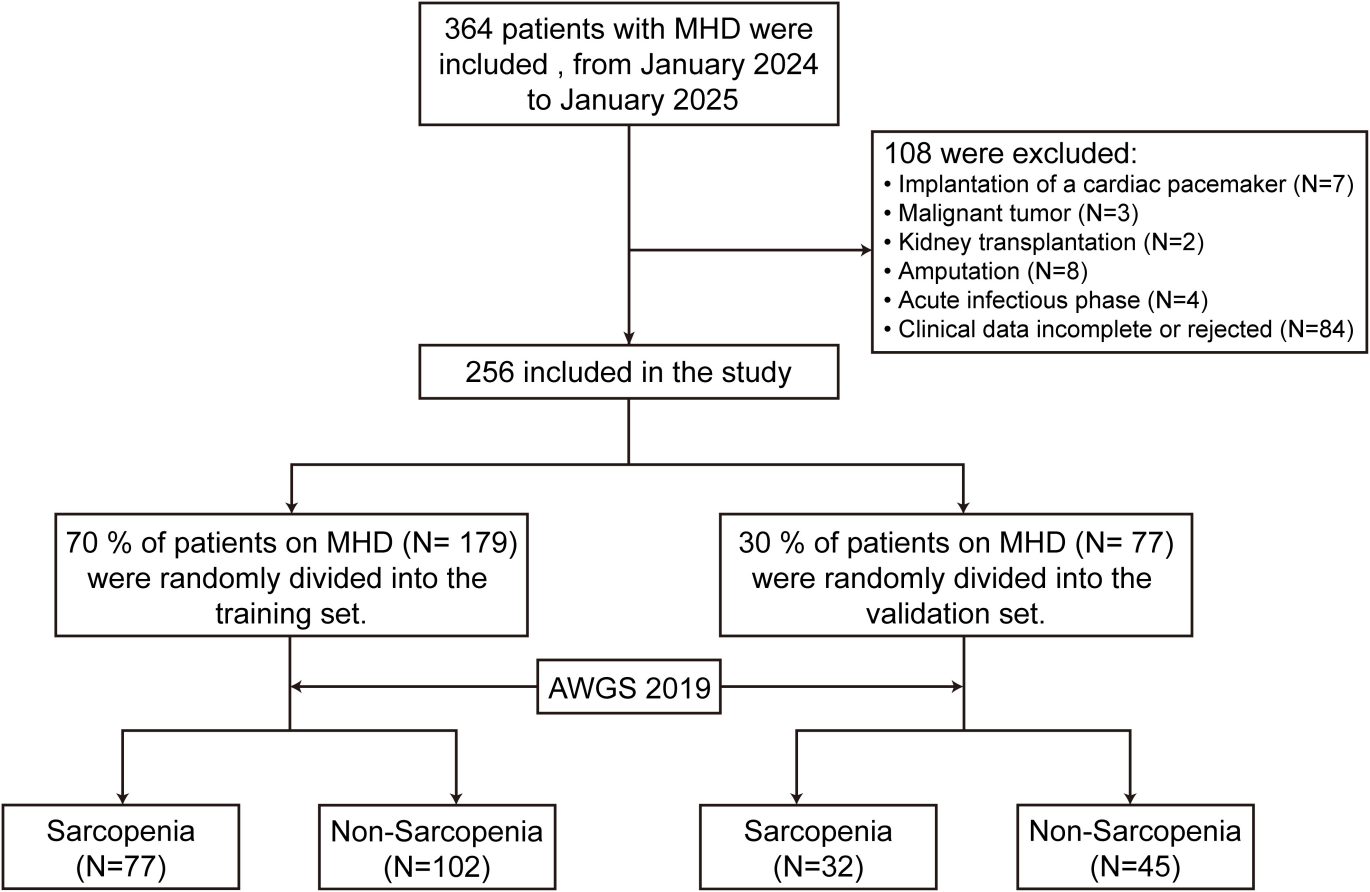
Study flowchart. MHD, maintenance hemodialysis; AWGS, asian working group for sarcopenia.

A total of 256 MHD patients were included in the study (Figure 1), with 139 males (54.2%) and 117 females (45.8%). Among the participants, 109 patients were diagnosed with sarcopenia (42.6%). The study population was randomly divided into a training set (*n* = 179) and a validation set (*n* = 77) at a 7:3 ratio. No significant differences were observed between the two groups in terms of demographic characteristics and major laboratory indicators (*p* > 0.05), ensuring a balanced data distribution (Table 1).

**TABLE 1:** Baseline characteristics of the training and validation sets.

Variables	Overall (*n* = 256)	Training set (*n* = 179)	Validation set (*n* = 77)	*P*-value
Diagnosis, n(%)				0.829
Non-Sarcopenia	147(57.422)	102(56.983)	45(58.442)	
Sarcopenia	109(42.578)	77(43.017)	32(41.558)	
Gender, n(%)				0.188
Female	117(45.703)	77(43.017)	40(51.948)	
Male	139(54.297)	102(56.983)	37(48.052)	
Hypertension, n(%)				0.44
No	81(31.641)	54(30.168)	27(35.065)	
Yes	175(68.359)	125(69.832)	50(64.935)	
Diabetes, n(%)				0.837
No	162(63.281)	114(63.687)	48(62.338)	
Yes	94(36.719)	65(36.313)	29(37.662)	
Cardiovascular diseases, n(%)				0.782
No	212(82.813)	149(83.240)	63(81.818)	
Yes	44(17.188)	30(16.760)	14(18.182)	
Age (years), median (IQR)	59(49, 70)	59(48, 69)	59(49, 70)	0.924
BMI (kg/m^2^), median (IQR)	22.9(20.9, 25.5)	23(20.9, 25.5)	22.7(20.8, 25.6)	0.539
Grip strength (kg), median (IQR)	17.9(12.7, 23.3)	18.1(12.8, 23.9)	17(12.5, 20.6)	0.492
Walk speed (m/s), mean (± SD)	0.958 ± 0.229	0.962 ± 0.228	0.949 ± 0.230	0.687
Dialysis vintage (months), median (IQR)	43(21, 73)	42(21, 73)	45(21, 75)	0.611
SM I (kg/m^2^), mean (± SD)	6.275 ± 1.136	6.293 ± 1.180	6.235 ± 1.026	0.709
Kt/V, median (IQR)	1.31(1.26, 1.36)	1.31(1.25, 1.36)	1.31(1.28, 1.36)	0.92
eGFR (mL/min/1.73 m^2^), median (IQR)	6.566(5.752, 8.051)	6.725(5.735, 8.115)	6.346(5.777, 7.891)	0.353
Creatinine (mg/dL), mean (± SD)	15.500 ± 5.202	15.442 ± 5.465	15.636 ± 4.528	0.785
CysC (mg/L), median (IQR)	6.28(5.48, 7.07)	6.24(5.45, 7.07)	6.39(5.53, 7.05)	0.507
Uric acid (μmol/L), median (IQR)	414.9(349.8, 487.5)	398.0(349.4, 489.0)	426.4(359.5, 477.7)	0.477
LVEF, median (IQR)	65(61, 69)	65(61, 69)	65(60, 70)	0.914
Hb (g/L), median (IQR)	110(96, 122)	110(94, 121)	110(100, 123)	0.463
Albumin (g/L), median (IQR)	40.1(37.1, 42.0)	40.1(36.7, 42.0)	39.8(37.4, 42.1)	0.891
Prealbumin (mg/L), mean (± SD)	311.621 ± 96.345	306.743 ± 93.448	322.961 ± 101.866	0.218
PTH (pg/mL), median (IQR)	236.0(113.0, 428.4)	233.0(121.0, 404.0)	236.0(108.3, 461.1)	0.804
NLR, median (IQR)	4.169(3.035, 6.381)	4.229(3.077, 6.958)	4.020(3.006, 5.963)	0.232
hs-CRP (mg/L), median (IQR)	2.06(0.95, 6.10)	1.76(0.92, 6.21)	2.48(1.01, 5.73)	0.358
ALP (U/L), median (IQR)	89.30(73.50, 108.00)	89.80(71.60, 108.00)	88.06(75.14, 109.00)	0.961
CK (U/L), median (IQR)	78.34(59.70, 108.00)	79.03(60.92, 108.35)	73.99(58.67, 106.61)	0.622
25(OH)D3 (ng/mL), median (IQR)	18.20(15.00, 23.77)	17.70(14.88, 23.77)	18.90(15.20, 24.51)	0.303
Glucose (mmol/L), median (IQR)	7.15(5.48, 9.64)	7.12(5.45, 9.83)	7.17(5.51, 9.31)	0.962
TG (mmol/L), median (IQR)	1.65(1.14, 2.75)	1.58(1.13, 2.65)	1.76(1.14, 2.81)	0.371
TC (mmol/L), median (IQR)	3.72(3.14, 4.42)	3.71(3.11, 4.40)	3.72(3.16, 4.46)	0.566
HDL-C (mmol/L), median (IQR)	0.99(0.81, 1.18)	0.98(0.81, 1.16)	1.01(0.80, 1.24)	0.515
LDL-C (mmol/L), median (IQR)	2.00(1.56, 2.43)	2.04(1.59, 2.43)	1.92(1.50, 2.49)	0.718
Calcium (mmol/L), mean (± SD)	2.139 ± 0.190	2.128 ± 0.188	2.166 ± 0.192	0.142
Phosphorus (mmol/L), median (IQR)	1.64(1.33, 2.08)	1.64(1.33, 2.11)	1.64(1.32, 2.03)	0.736
Magnesium (mmol/L), mean (± SD)	1.068 ± 0.177	1.067 ± 0.174	1.071 ± 0.184	0.853
CO2CP (mmol/L), median (IQR)	19.4(17.2, 21.2)	19.3(17.2, 21.2)	20.0(17.2, 21.4)	0.565

### 3.2 Feature selection

**FIGURE 2:**
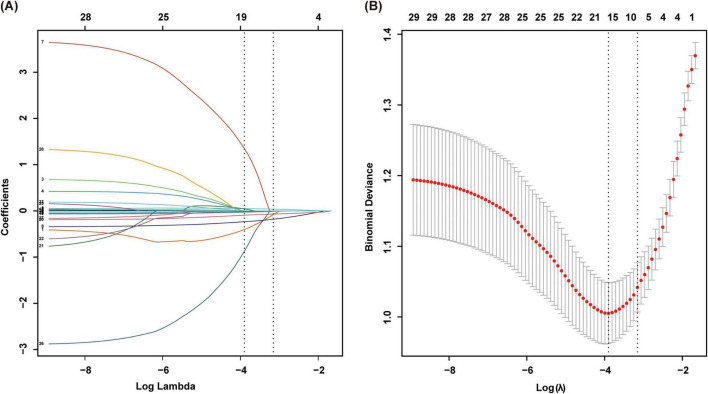
Variable selection using LASSO regression. **(A)** Path plot of LASSO regression coefficients, with 10-fold cross-validation used. Vertical lines indicate the selected values where the optimal lambda value results in eight non-zero coefficients. **(B)** Cross-validation curve of LASSO regression. Vertical dashed lines indicate the minimum mean squared error (λ = 0.02) and minimum distance standard error (λ = 0.043).

Key features were initially selected using LASSO regression with 10-fold cross-validation. An optimal λ value (λ = 0.043) was determined, selecting eight candidate features (Figure 2). To control for confounding factors, multivariate logistic regression was performed, identifying seven significant predictors: gender, age, BMI, 25(OH)D3, LVEF, Cr, and eGFR (Table 2). While many other nutritional and biochemical indicators showed potential associations in univariate analysis, they did not provide additional independent predictive value in the multivariate framework. These variables were significantly associated with sarcopenia risk (*p* < 0.05), providing a solid foundation for model construction.

**TABLE 2:** Multivariable logistic regression analysis.

Variables	B	Wald	*P*-value*	OR (95%CI)
Gender	0.712	3.888	0.049	2.038(1.004∼4.134)
Age	0.064	14.128	0.000	1.067(1.031∼1.103)
BMI	−0.323	26.077	0.000	0.724(0.640∼0.820)
25(OH)D3	−0.058	6.596	0.010	0.944(0.903∼0.986)
LVEF	−0.052	4.770	0.029	0.949(0.906∼0.995)
Cr	−0.144	9.152	0.002	0.866(0.789∼0.951)
eGFR	−0.186	4.240	0.039	0.830(0.695∼0.991)
(Intercept)	12.167	20.880	0.000	–

*p < 0.05.

### 3.3 Model performance comparison

**FIGURE 3:**
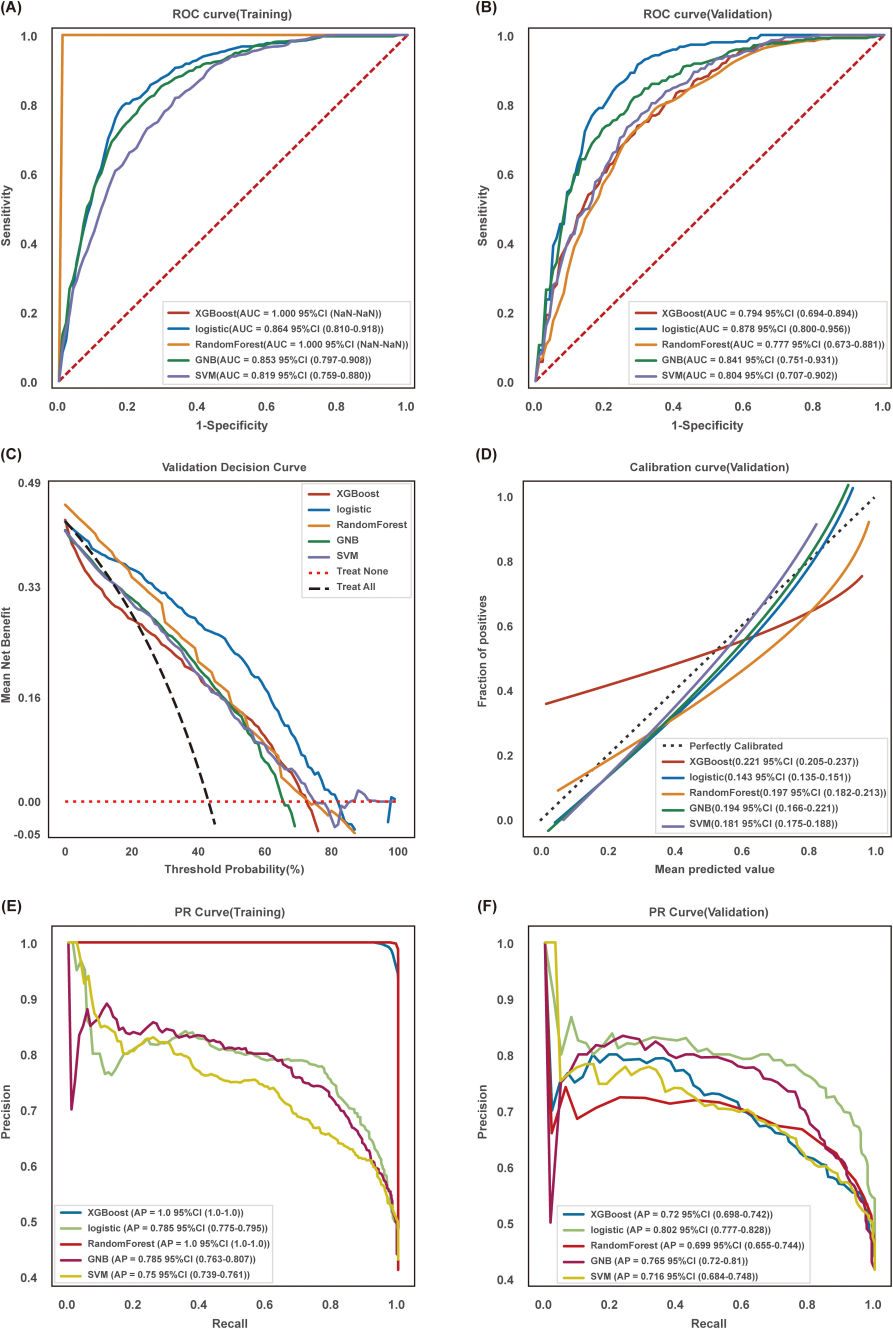
Comprehensive analysis of machine learning models. **(A)** AUC for the training set and **(B)** the validation set. **(C)** Decision curve analysis (DCA) for the validation set, where the black dashed line represents the hypothesis that all patients have sarcopenia, and the red dashed line represents the hypothesis that no patients have sarcopenia. **(D)** Calibration curve for the validation set, with the x-axis representing the average predicted probability and the y-axis representing the actual probability of events. The dashed diagonal line serves as the reference, and the smooth solid lines represent the model fit lines for different models. The closer the fit line is to the reference line, the smaller the value in parentheses, indicating more accurate model predictions. **(E)** PR curve and AP value for the training set. **(F)** The validation set. The y-axis represents precision and the x-axis represents recall. Different colors in the figure correspond to the respective models, with values presented as mean and 95% CI.

The performance of five machine learning models—XGBoost, Logistic Regression, Random Forest, SVM, and GNB—was evaluated: (1) AUC Analysis: The Logistic Regression model performed best in the validation set (AUC = 0.878, 95% CI: 0.800–0.956), while XGBoost and Random Forest exhibited better performance in the training set (Figures 3A,B). (2) Decision Curve Analysis (DCA): Across a wide range of risk thresholds, the Logistic Regression model demonstrated the best clinical applicability (Figure 3C). (3) Calibration Curve: The predicted values of the Logistic Regression model were highly consistent with the actual values, indicating good calibration (Figure 3D). (4) Precision-Recall (PR) Curve: The Logistic Regression model had the highest average precision (AP value), further confirming its reliability (Figures 3E,F). Based on overall performance, the Logistic Regression model was selected as the optimal model.

### 3.4 Stability and generalization ability of the optimal model

**FIGURE 4:**
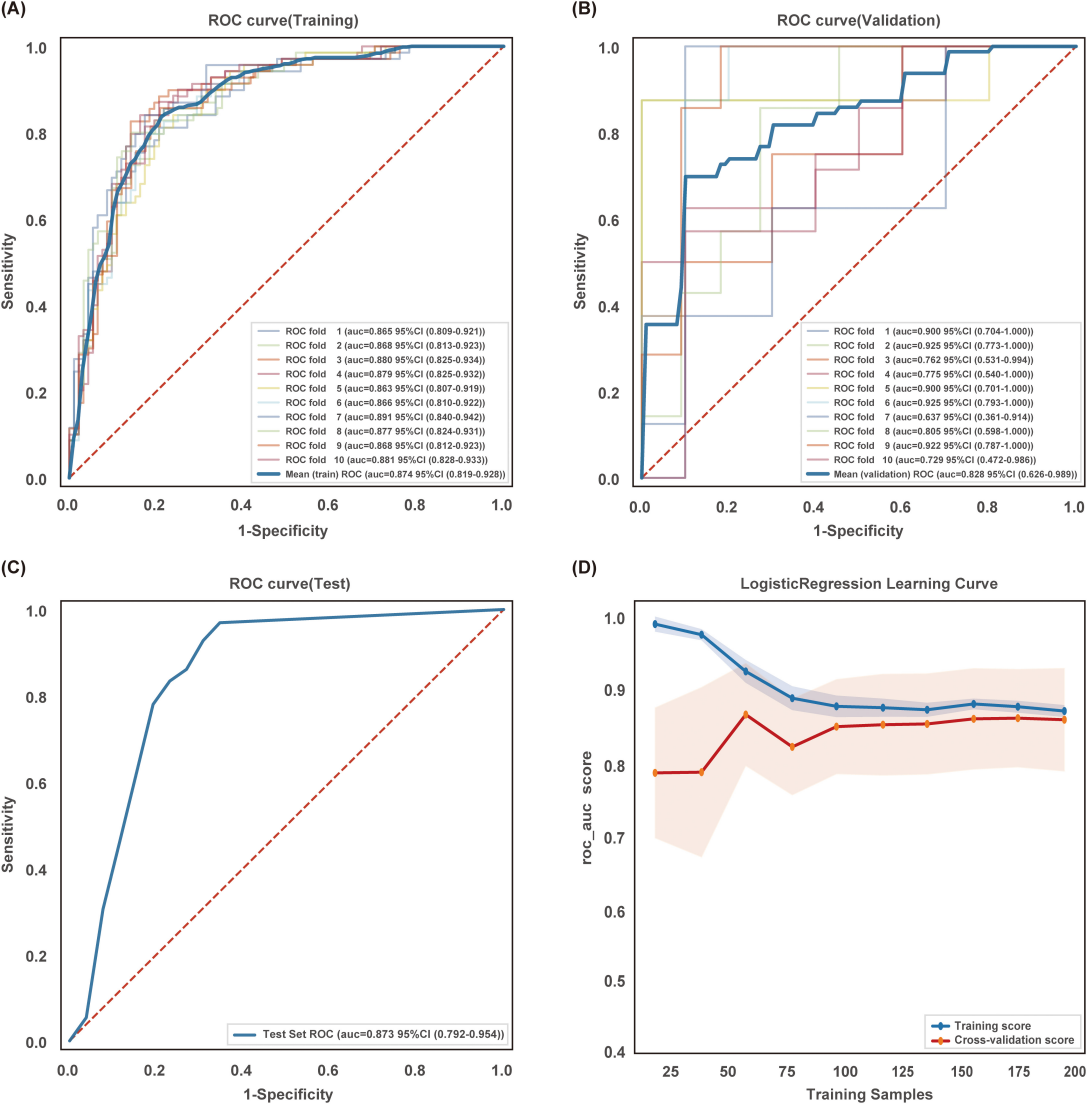
Training, validation, and testing of the logistic regression model. **(A)** ROC curve and AUC for the training set and **(B)** the validation set. Solid lines of different colors represent the results of 10-fold cross-validation. **(C)** ROC curve and AUC for the test set. **(D)** Learning curve. The solid blue line represents the training set, and the solid red line represents the cross-validation set. Each point represents the mean AUC (± SD), and the shaded areas represent the 95% confidence intervals. The curves converge with a small gap, indicating good generalization and model stability.

The Logistic Regression model demonstrated robust performance across all datasets. During 10-fold cross-validation, it achieved a mean AUC of 0.874 (95% CI: 0.819–0.928) on the training set and 0.828 (95% CI: 0.626–0.989) on the validation set. Notably, its generalizability was confirmed on an independent hold-out test set, with an AUC of 0.873 (95% CI: 0.792–0.954) (Figures 4A–C). Learning curve analysis revealed converging training and cross-validation curves with a narrow, stable gap, indicating effective generalization without overfitting. Each point on the curves represents the mean AUC (± SD) from 10 repetitions, with shaded areas denoting the 95% confidence intervals (Figure 4D). The curves demonstrate that as the training data volume increased, both training and cross-validation performance metrics stabilized, further affirming the model’s consistent generalization capability across varying sample sizes. Collectively, these results underscore the model’s performance robustness and stability.

### 3.5 Model interpretability analysis

**FIGURE 5:**
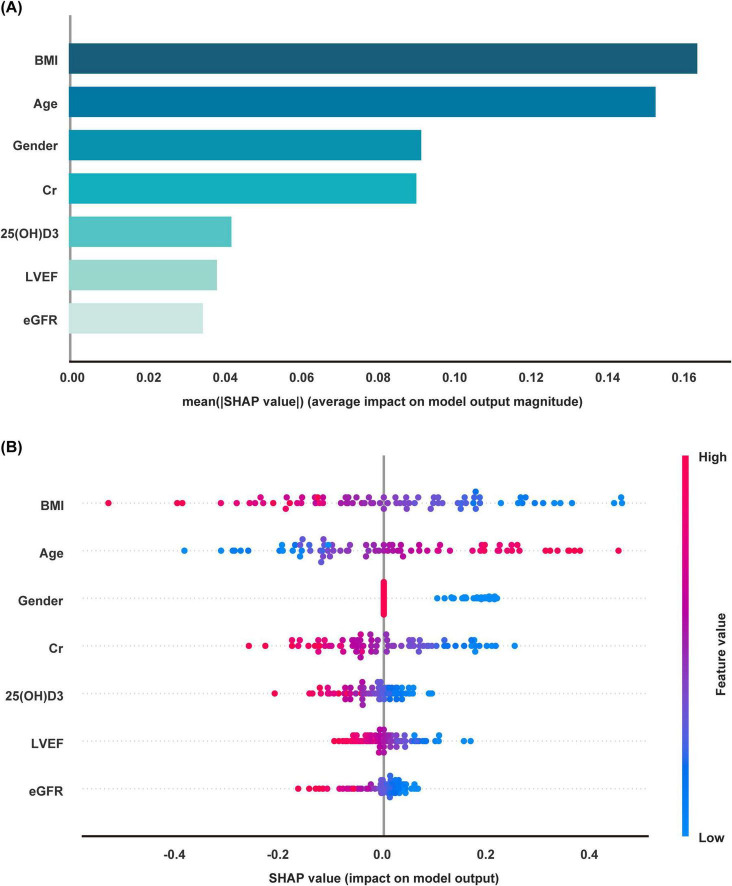
SHAP model interpretation. **(A)** Feature importance ranking. The matrix plot displays the importance of each covariate in the final prediction model. **(B)** SHAP summary plot showing the distribution of SHAP values for each feature. Each point represents the SHAP value of a feature for each patient, with the x-axis representing the SHAP value. The color gradient from red to blue indicates the feature value, from high to low.

**FIGURE 6:**
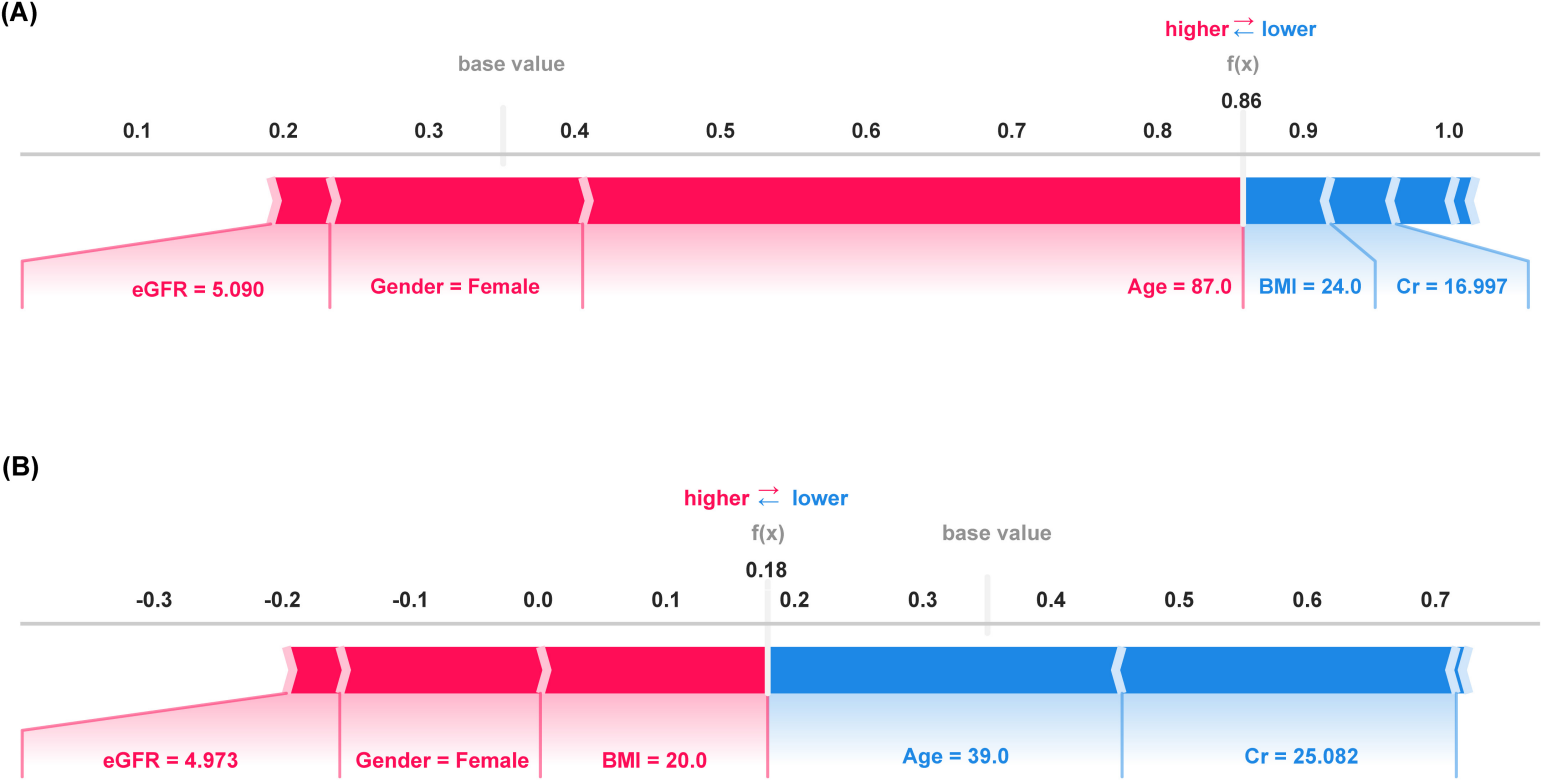
SHAP model interpretation for individual predictions. **(A)** SHAP force plot for patient A (true positive) and **(B)** SHAP force plot for patient B (True Negative). The length of the color bar represents the contribution value, with red indicating a positive contribution and blue indicating a negative contribution.

SHAP was employed for the interpretability analysis of the Logistic Regression model: (1) Feature Importance Ranking: BMI was the most significant predictor for sarcopenia risk, followed by age, gender, Cr, 25(OH)D3, LVEF, and eGFR (Figure 5A). (2) Feature Direction of Effect: High BMI, high 25(OH)D3 levels, and younger age were protective factors for sarcopenia, whereas low Cr, LVEF, eGFR, and female gender significantly increased sarcopenia risk (Figure 5B). (3) Personalized Risk Assessment: SHAP analysis provided intuitive visualizations of feature contributions for individual patients. For example, for Patient A (true positive), the model predicted an 86.0% probability of sarcopenia occurrence, with older age, female gender, and low eGFR as the primary risk factors (Figure 6A). For patient B (true negative), the model predicted an 18.0% probability of sarcopenia, with young age and higher Cr levels as the main protective factors (Figure 6B).

## 4 Discussion

In recent years, the development of risk prediction models for sarcopenia in dialysis patients has advanced considerably. Various machine learning models, including Logistic Regression, Random Forest, and SVM, have been widely applied (18, 19). However, most existing studies primarily focus on improving prediction performance, with insufficient exploration of the model’s decision-making mechanisms. Notably, few studies have systematically integrated advanced techniques such as SHAP to interpret key risk features.

To address this gap, we present a framework that integrates the clinically interpretable Logistic Regression model with SHAP analysis for sarcopenia prediction in patients undergoing MHD. This approach not only ensures model performance (AUC = 0.828) but also utilizes SHAP to provide individualized predictive insights, significantly enhancing both clinical interpretability and practical utility. Moreover, the clinical indicators employed in this study—such as BMI, age, and 25(OH)D3—are routine and easily obtainable, further improving the model’s applicability and feasibility in real-world medical settings.

### 4.1 Clinical significance of key predictors

#### 4.1.1 BMI

BMI emerged as one of the most important predictors in this study, aligning with previous research (20). Lower BMI is closely associated with muscle wasting and malnutrition in MHD patients (17), thereby increasing the risk of all-cause mortality and cardiovascular events. A meta-analysis of 12 studies found that BMI is one of the most common predictor in models, with AUC values exceeding 0.7 (21). However, BMI is not a perfect metric, especially in MHD patients, as body composition may change due to dialysis and malnutrition, potentially masking the true risk of muscle loss. For instance, although BMI may appear normal, an increase in body fat could result in falsely normal values, thus underestimating muscle loss risk (22). Therefore, clinical practice should combine other biochemical indicators (such as creatinine and LVEF) to provide a more comprehensive assessment of muscle status.

#### 4.1.2 Age and gender

Age and gender are important predictors of sarcopenia, particularly in MHD patients, where age is a significant risk factor (23, 24). As age increases, muscle mass and strength decline, a trend that is particularly evident in dialysis patients. Additionally, gender differences cannot be overlooked; studies indicate that women are 20% more likely to develop sarcopenia than men (25). In our study, the overall prevalence of sarcopenia was 42.6%, with 47.7% of men and 52.3% of women affected. Research suggests that testosterone promotes muscle synthesis, increasing muscle size and strength (26). Similarly, estrogen helps regulate protein synthesis in skeletal muscle (27). However, the impact of estrogen on muscle remains under investigation, with insufficient evidence to suggest a significant effect (28). These age and gender differences highlight the need for personalized strategies in clinical interventions. For example, for female patients, particularly in the elderly population, nutritional assessments and rehabilitation training should be prioritized to reduce the incidence of sarcopenia.

#### 4.1.3 Renal function indicators (Cr and eGFR)

Creatinine and eGFR were identified as important predictors for sarcopenia in this study. Creatinine serves as a direct indicator of muscle metabolism, and its decreased concentration typically reflects muscle mass loss (29). In MHD patients, creatinine levels, influenced by kidney function, can be an important marker for muscle wasting (30). The decline in eGFR is commonly associated with the progression of kidney dysfunction, which may, through chronic inflammation and metabolic disturbances, accelerate the onset of sarcopenia (31).

However, we acknowledge that the reliability of eGFR in end-stage renal disease (ESRD) may be compromised. Altered renal clearance and chronic inflammation, both prevalent in MHD patients, can affect the accuracy of eGFR measurements. To address these limitations, we employed the CKD-EPI cystatin C-based equation for eGFR calculation. Cystatin C is less affected by muscle mass compared to creatinine, making it a potentially more reliable indicator of renal function in this patient population.

Despite the improvements afforded by the CKD-EPI cystatin C equation, we recognize that eGFR remains a complex predictor of sarcopenia. Future research should consider additional biomarkers, such as the Cr/Cys-C ratio, to enhance the specificity and accuracy of sarcopenia predictions. Integrating these markers could refine the prediction models further. For the present analysis, the use of the CKD-EPI cystatin C equation allows for a more tailored assessment of kidney function in MHD patients and remains a critical variable in our sarcopenia prediction model.

#### 4.1.4 Vitamin D

Vitamin D was shown to be a protective factor for sarcopenia in this study. Vitamin D significantly lays a significant role in bone health, immune regulation, and muscle function, all of which are critical in the development of sarcopenia (32). Our study found that higher 25-hydroxyvitamin D3 levels effectively slowed muscle atrophy, particularly in MHD patients, where vitamin D supplementation is considered an effective and simple intervention (33). Vitamin D exerts its protective effect by inhibiting the renin-angiotensin system (RAS) (34). Specifically, 1,25-dihydroxyvitamin D_3_ activates the vitamin D receptor (VDR) in skeletal muscle cells, which directly suppresses renin gene transcription and reduces the production of angiotensin II (Ang II). Ang II, a potent inducer of muscle catabolism, promotes oxidative stress, inflammation, and activation of the ubiquitin-proteasome system (UPS), leading to muscle protein degradation (35). By downregulating Ang II levels, vitamin D mitigates these catabolic effects, preserves mitochondrial function, and promotes protein synthesis in muscle cells. This mechanism is particularly crucial for MHD patients, who are often deficient in vitamin D and experience elevated RAS activity, both of which accelerate sarcopenia progression. Therefore, vitamin D supplementation should be routinely incorporated into the management of MHD patients to prevent muscle loss and improve overall muscle health.

#### 4.1.5 Heart function indicators

LVEF, a crucial indicator of heart function, was identified as a significant predictor for sarcopenia in this study. Reduced LVEF is often associated with inadequate systemic blood perfusion and tissue hypoxia, which can negatively affect skeletal muscle mass and function (36). Studies have shown that patients with reduced LVEF often exhibit more pronounced muscle wasting (37). Additionally, the presence of sarcopenia significantly increases the risk of major adverse cardiovascular events (MACE) in MHD patients (38). This study further validated the effectiveness of LVEF as a predictor of sarcopenia and suggested that clinicians should regularly monitor heart function, particularly in high-risk sarcopenia groups, to provide dual intervention opportunities for patients.

### 4.2 Clinical value of interpretable machine learning

In this study, comparative evaluation of multiple machine learning models identified Logistic Regression as the optimal choice for predicting sarcopenia risk in MHD patients. Building on its inherent interpretability, we applied SHAP to further elucidate the contributions of key features, thereby enhancing the model’s transparency and practical clinical value. SHAP visualizations, which rank feature contributions, assist physicians in understanding how various variables influence the occurrence of sarcopenia, thereby supporting precise and informed clinical decision-making.

### 4.3 Clinical application prospects

The model developed in this study has significant clinical application potential. First, based on routine clinical indicators [such as BMI, Cr, 25(OH)D3, etc.], the model can rapidly identify high-risk sarcopenia patients, making it particularly suitable for resource-limited primary healthcare settings. Second, when combined with SHAP analysis, the model supports personalized risk assessments, helping to devise tailored intervention strategies. Finally, the model’s simple structure facilitates its integration into electronic health record systems, offering broad potential for widespread adoption.

### 4.4 Study limitations and future directions

Despite the significant progress made in this study, the following limitations must be considered: (1) The single-center, retrospective design and relatively small sample size (*n* = 256) may introduce selection bias, potentially affecting the model’s generalizability and stability. A small sample size may lead to overoptimistic performance estimates, reducing the robustness of the model when applied to diverse patient populations. To address this, future studies should incorporate multicenter prospective validation with larger sample sizes to validate the model’s performance across a broader, independent cohort. (2) This study did not account for the dynamic progression of sarcopenia, and future research should include longitudinal follow-up data to assess the model’s predictive ability as the disease progression. (3) Since SHAP relies on statistical correlations, it may not fully capture causal relationships. Therefore, future research should combine biological experiments to better understand the mechanisms behind the predictive factors.

Future studies could further optimize and expand the model by: (1) Mitigating Optimism Bias: To address optimism bias, we plan to apply bootstrap resampling in subsequent analyses. This technique will allow for a more accurate quantification of the optimism bias and help adjust the model’s performance estimates. Additionally, multicenter prospective validation will be incorporated to assess the model’s generalizability across diverse clinical settings and patient populations. (2) Incorporating Multi-Omics Data: We aim to integrate multi-omics data (e.g., genomics, metabolomics) to capture a broader range of sarcopenia risk factors. This will enhance the model’s prediction accuracy by incorporating biological insights that may not be captured by traditional clinical data alone. (3) Exploring Deep Learning Models: To further enhance prediction performance, we plan to explore deep learning models. These models may uncover more complex patterns within the data, and integrating advanced interpretability tools will help maintain the model’s clinical usefulness while ensuring transparency in predictions.

## 5 Conclusion

This study developed a Logistic Regression model combined with SHAP analysis, demonstrating its superior performance in predicting sarcopenia risk in MHD patients. The model offers high interpretability and easy feature accessibility, providing a valuable tool for early screening and personalized interventions for sarcopenia. Although further validation is required, the findings of this study lay a solid foundation for the precise management of sarcopenia and are expected to improve the long-term prognosis of MHD patients.

## Data Availability

The raw data supporting the conclusions of this article will be made available by the authors, without undue reservation.
